# Residual Postoperative Valgus After Total Knee Arthroplasty for Preoperative Valgus Deformity Results in Inferior Patient-Reported Outcomes

**DOI:** 10.1016/j.artd.2025.101660

**Published:** 2025-03-13

**Authors:** Anastasia A. Hunt, Ian Hollyer, Nicole S. Pham, William J. Maloney, James I. Huddleston

**Affiliations:** Department of Orthopaedic Surgery, Stanford University School of Medicine, Redwood City, CA, USA

**Keywords:** Valgus alignment, Mechanical alignment, Total knee arthroplasty, Patient-reported outcomes

## Abstract

**Background:**

The optimal postoperative coronal alignment after total knee arthroplasty (TKA) for preoperative valgus deformity is unknown. This study aims to evaluate the impact of postoperative coronal alignment on clinical outcomes following TKA for valgus deformities.

**Methods:**

Patients in preoperative valgus undergoing primary TKA between 2010 and 2020 with at least 1 year of follow-up were retrospectively reviewed. Preoperative and postoperative mechanical alignment was assessed on long-leg radiographs via the hip-knee-ankle angle. Postoperative alignment was categorized into valgus (>2° valgus), neutral (within 2° of the mechanical axis), or varus (>2° varus). Patient demographics, preoperative and postoperative outcome scores, and complications were collected.

**Results:**

106 patients met inclusion criteria, with a mean preoperative valgus deformity of 11° (standard deviation ± 6.1). Postoperatively, 18 patients were in varus alignment, 58 were neutral, and 30 remained in valgus. At 2-year follow-up, multivariate analyses showed that patients in neutral or varus alignment postoperatively had superior Veterans RAND 12-Item Health Survey Physical and Knee injury and Osteoarthritis Outcome Score Pain scores compared to those in residual valgus. Varus knees showed significantly greater improvement in Knee Society Score Function scores compared to valgus knees. At final follow-up, Knee injury and Osteoarthritis Outcome Score Pain scores were significantly better in varus knees. Patients in varus were 7.79 times more likely to achieve the minimal clinically important difference VR-12 Physical score, while patients in neutral were 3.26 times more likely to achieve the minimal clinically important difference for Knee Society Score Function when compared to valgus knees.

**Conclusions:**

Correcting preoperative valgus coronal alignment to neutral or varus yields improved patient-reported outcomes when compared to residual valgus.

## Introduction

Total knee arthroplasty (TKA) is an effective treatment for end-stage osteoarthritis as it provides pain relief, restores function, and maintains excellent long-term survivorship [1]. Coronal plane deformities are central in surgical decision-making when determining optimal implant positioning and may impact implant survivorship. Most arthritic knees present with varus deformities, but approximately 10%-15% of knees are in valgus alignment preoperatively [2]. Patient satisfaction may be higher in TKAs with residual coronal plane deformity than in those restored to neutral mechanical alignment [3]. However, current American Academy of Orthopedic Surgeons guidelines state that there is “no difference in composite outcomes, functional outcomes, or complications between kinematic and mechanical alignment principles in TKA” [4,5].

The literature assessing the optimal postoperative coronal plane alignment for patients with preoperative valgus deformities is not robust [6,7]. In 2018, Lee et al. retrospectively assessed a single surgeon database of 94 patients undergoing TKA between 2005 and 2015 with at least 24 months of follow-up. Of these, 69 patients were placed into neutral alignment postoperatively (0 ± 3°), 17 into mild valgus (3°-6°), and 7 into severe valgus (>6°). The study found no differences in Western Ontario and McMaster Universities Osteoarthritis Index scores or Knee Society Scores (KSS) between postoperative alignment groups [8]. Notably, this study included 20 patients who underwent bilateral TKA, with one side selected randomly for inclusion. This selection methodology may have confounded the analysis as there is some evidence that satisfaction can differ between the first and second TKA [9]. Given the necessity for additional studies to inform surgical decision-making, this study aimed to assess the effects of postoperative coronal plane alignment after TKA on patients with preoperative valgus deformity. Specifically, this study evaluated whether patient-reported outcomes were superior with residual postoperative valgus alignment or correction to neutral or varus.

## Material and methods

After receiving Institutional Review Board approval, patients who underwent primary TKA with a gap-balancing technique by 1 of 4 fellowship-trained arthroplasty surgeons at a single tertiary academic hospital between January 2010 and December 2020 were reviewed. Patients aged more than 18 years with preoperative valgus coronal alignment and at least 1 year of follow-up were included. Preoperatively and at each follow-up visit, patient-reported outcome measures (PROMs) were completed and stored in an institutional registry. These included the University of California Los Angeles Activity Scale (UCLA), Veterans RAND 12-Item Health Survey (VR-12), Knee injury and Osteoarthritis Outcome Score (KOOS), Knee injury and Osteoarthritis Outcome Score for Joint Replacement, and the KSS.

Exclusion criteria were revision TKA, less than 1 year of follow-up postoperatively, varus or neutral preoperative coronal alignment, or previous arthroplasty on the involved knee (eg, conversion from unicompartmental knee arthroplasty). Patients were also excluded if they did not have at least 1 preoperative and postoperative PROM within the registry. Demographic data, including age, sex, body mass index, American Society of Anesthesiologists physical status classification, implant type, and revision for any cause were collected.

All patients received standard 3-view (anteroposterior, lateral, and patella axial) preoperative and postoperative short-knee and standing long-leg radiographs. We defined valgus alignment as >2° of valgus from the mechanical axis, neutral alignment within 2° of the mechanical axis, and varus alignment as >2° of varus from the mechanical axis. All radiographic measurements were performed by 2 individuals (A. A. H. and I. H.). The mechanical axis was assessed using the hip-knee-ankle angle from the center of the femoral head to the intercondylar notch of the distal femur, to the center of the ankle joint. Images were viewed and measured using Sectra picture archiving (Sectra Medical, Linköping, Sweden) and communication system software (Picture Archiving and Communication System) (Linköping, Sweden).

### Patient demographics

**Figure 1:**
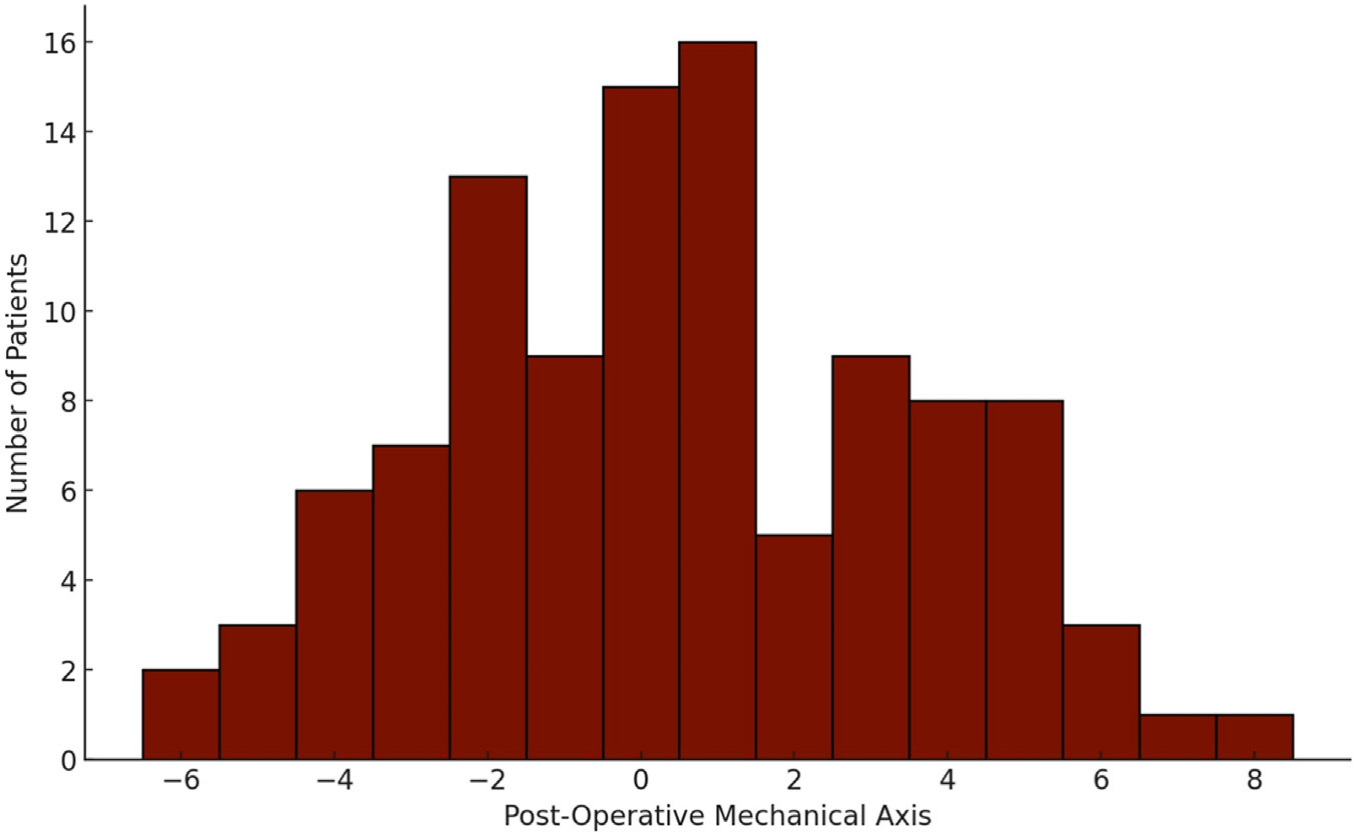
Histogram of postoperative alignment. Key: negative numbers on x-axis represent varus and positive numbers represent valgus.

Between January 2010 and December 2020, 2971 primary TKAs were performed. Of these, 431 were in valgus alignment preoperatively with at least 1 year of follow-up. One hundred and six of the 431 patients had preoperative and postoperative PROMs and were ultimately included in analyses. The cohort was predominantly female (69.8%), had a mean body mass index of 29.3 (standard deviation [SD] ± 5.8), and a mean preoperative valgus alignment of 11° (SD ± 6.1). Overall, 18 patients were corrected to varus postoperatively (17%), 30 (28.3%) remained in valgus, and 58 (54.5%) were restored to neutral. The varus group had a median alignment of 4° (3°-6°), the neutral group had a median alignment of 0° (2° varus-2° valgus), and the valgus group had a median postoperative alignment of 4° of valgus (3°-8°) (Fig. 1). The preoperative and postoperative alignment by surgeon can be found in Table 1. There were no significant differences in the severity of preoperative valgus alignment by surgeon (Table 1), or in demographic characteristics between the postoperative alignment groups (Table 2).

**Table 1:** Preoperative and postoperative patient alignment by surgeon.

Surgeon	N TKA	Mean preoperative valgus alignment	N patients postoperative valgus (%)	N patients postoperative neutral (%)	N patients postoperative varus (%)
1	11	9°	4 (36%)	6 (55%)	1 (9%)
2	44	9°	16 (36%)	25 (57%)	3 (7%)
3	15	8°	7 (47%)	8 (53%)	0 (0%)
4	36	9°	3 (8%)	19 (53%)	14 (39%)

N, number; TKA, total knee arthroplasty.

**Table 2:** Demographic and clinical differences between alignment groups.

Demographic and clinical variables	Postoperative alignment			*P* value
	Valgus (n = 30)	Neutral (n = 58)	Varus (n = 18)	
Age (mean, range)	69.5 (34-85)	68 (20-85)	68 (18-83)	.80
BMI (mean, range)	26.7 (20.4-30.5)	29 (19-41.1)	30.1 (19.5-43.7)	.30
Preoperative alignment (mean, range)	10 (3-33)	11 (1-25)	10.5 (3-38)	.77
Postoperative alignment (mean, range)	4 (3-8)	0 (−2 to 2)	−4 (−6 to −3)	**<.001**
Female (n, %)	25 (83)	39 (67)	10 (0.55)	.09
ASA classification (n, %)				
1	2 (7)	2 (3)	1 (6)	.69
2	13 (43)	29 (5)	6 (33)	
3	15 (50)	27 (47)	11 (61)	
Implant type (n, %)				
CCK	1 (3)	3 (5)	1 (5)	.50
Hinge	1 (3)	0 (0)	1 (5)	
CR + MC	4 (14)	6 (1)	0 (0)	
PS	23 (79)	49 (84)	16 (89)	
Revision (n, %)				
No	27 (90)	54 (93)	18 (100)	.42
Yes	3 (10)	4 (7)	0 (0)	
Reason for revisions (n, %)				
Infection	3 (10)	1 (2)	0 (0)	
Patellar maltracking	0 (0)	2 (3)	0 (0)	
Polywear	0 (0)	1 (2)	0 (0)	

ASA, American Society of Anesthesiologists; BMI, body mass index; CCK, constrained condylar knee; CR, cruciate retaining; MC, medial congruent; PS, posterior stabilized.

**Figure 2:**
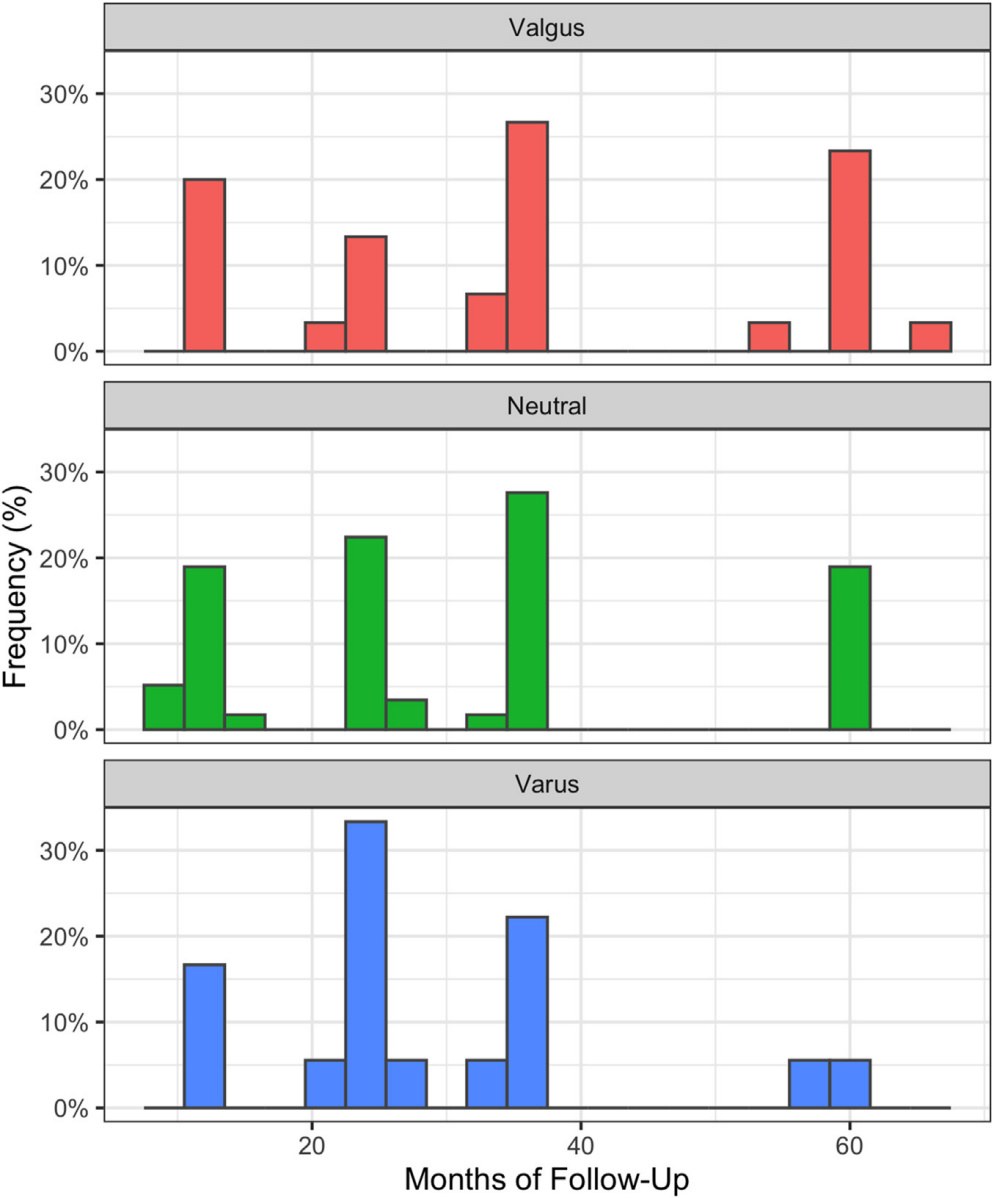
Distribution of final follow-up for each postoperative alignment group.

Eighty-four percent of patients received a posteriorly stabilized implant, 9.5% received a cruciate retaining or medial congruent implant, 4.7% received a cruciate condylar constrained implant, and 1.9% received a hinged implant. Seven patients (6.7%) underwent revision surgery—4 for infection, 2 for patellar maltracking, and 1 for polyethylene wear. There were no statistical differences in the revision rates between alignment groups, but no patients in postoperative varus underwent revision. Overall, 95 patients (89.6%) had 1-year follow-up, 75 patients (70.8%) had 2-year follow-up, and 22 patients (20.8%) had follow-up more than or equal to 5 years. The number of patients at 1-year, 2-year, and final follow-up within each postoperative alignment group can be found in Table 3. The median follow-up was 26 months with an interquartile range of 24-37 months. Final follow-up did not differ significantly between groups (Table 4, Fig. 2).

**Table 3:** Number of patients at each follow-up timepoint by postoperative alignment group.

Number of patients	Valgus (N = 30)	Neutral (N = 58)	Varus (N = 18)
1 year	28 (93%)	52 (90%)	15 (83%)
2 year	22 (73%)	39 (67%)	14 (78%)
Final follow-up	30 (100%)	58 (100%)	18 (100%)

**Table 4:** Final follow-up length by postoperative alignment group.

Final follow-up time (months)	Mean	SD	Range	*P* value
Valgus	36.4	18.3	11.9-65.8	.284
Neutral	31.6	16.9	9.3-61.0	
Varus	29.1	13.7	11.7-60.7	

SD, standard deviation.

### Data analysis

PROMs were analyzed at 1 and 2 years postoperatively, as well as at final follow-up. To define the minimal clinically important difference (MCID) for each PROM, we reviewed the literature to identify previously established values. For the KSS, the MCID is not universally agreed upon. One study calculated the MCID for KSS to be 7.2 points using anchor-based and distribution-based methods [10]. A receiver operating characteristic curve analysis showed that the cutoff point was 8.9 with an area under the curve of 0.75 [10]. Thus, we used a value of 7.2 for our analyses. For the VR-12, there is no agreed-upon value for TKA, but recent studies in shoulder arthroplasty and sports medicine have reported an MCID of 4.94 for the VR-12 Physical and 5.99 for the VR-12 Mental [[11], [12], [13]]. Given these previous studies, we set the MCID for VR-12 Physical at 4.94 and VR-12 Mental at 5.99. Similarly, for the KOOS and Knee injury and Osteoarthritis Outcome Score for Joint Replacement, the MCID has yet to be established in TKA. However, previous research has suggested that an MCID of 8-10 is appropriate for the KOOS [14,15]. Thus, we chose our KOOS MCID as 10. Finally, several studies have previously used an MCID of 0.92 for UCLA [16,17]. Thus, we used a UCLA MCID of 0.92.

A power analysis was performed prior to the initiation of this study. An a-priori calculation determined that 115 encounters per alignment group would provide >90% power to detect differences in the change from baseline equal to or more than the MCIDs for the UCLA, VR-12, KOOS, and KSS PROMs via single-factor analysis of variance with a 2-sided significance of 0.05. Pairwise post-hoc tests were adjusted for multiple comparisons with the Tukey-Kramer method. These calculations were performed based on the variability in these measures observed in similar populations, with the largest sample size selected for conservatism.

Descriptive statistics were presented as means and SDs for continuous variables and counts and percentages for categorical variables. Kruskal-Wallis, Chi-square, and Fisher’s exact tests evaluated demographic and clinical differences among alignment groups. After controlling for follow-up length and preoperative PROM score, analysis of variance tests and multivariable linear and logistic mixed-effects regression models were used to evaluate differences in postoperative PROMs, changes in PROMs from baseline, and the odds of MCID achievement among alignment groups at 2 years postoperatively and final follow-up. Chi-square and Fisher’s exact tests evaluated differences in MCID achievement rates among alignment groups. If significant, *post-hoc* pairwise tests reported Tukey-adjusted and false discovery rate-adjusted *P* values. All analyses were completed in RStudio version 2023.03.1+446 (Posit PBC, Boston, MA) using a 2-sided significance level of 0.05.

## Results

### Improvements in PROMs

Univariate analyses did not demonstrate significant differences in PROMs between postoperative alignment groups at 1-year or 2-year follow-up (Table 5). At 1-year follow-up, multivariate analyses demonstrated that patients in neutral postoperative alignment had lower improvements in VR-12 Mental scores compared to patients in residual valgus by 3.6 points (*P* = .037; 95% confidence interval [CI]: −6.9 to −0.2). At 2 years, multivariate analyses showed that patients in neutral or varus postoperative alignment had greater improvements in VR-12 Physical scores compared to patients in residual valgus by 5.7 (*P* = .033; 95% CI: 0.5-11.0) and 7.6 points (*P* = .031; 95% CI: 0.7-14.4), respectively. Neutral and varus knees showed greater improvements in KOOS Pain scores compared to valgus knees by 10 (*P* = .015; 95% CI: 2.0-18) and 11.6 points (*P* = .034; 95% CI: 0.9-22.3), respectively. Varus knees also had a significantly greater average improvement in KSS Function compared to valgus by 12.5 points (*P* = .024; 95% CI: 1.7-23.3; Table 6).

**Table 5:** Univariate analysis of change from preoperative PROM.

ΔPROM per alignment group	1-year follow-up		2-year follow-up		Final follow-up			
	Mean (95% CI)	*P* value	Mean (95% CI)	*P* value	Mean (95% CI)	*P* value	Pairwise	Tukey adj. *P* value
△UCLA								
Valgus	0.7 (0.1-1.3)	.620	0.5 (−0.23 to 1.23)	.911	0.3 (−0.32 to 0.92)	.610		
Neutral	0.5 (0.0-1.0)		0.7 (0.2-1.2)		0.7 (0.19-1.21)			
Varus	1.0 (−0.1 to 2.1)		0.7 (−0.45 to 1.85)		0.7 (−0.27 to 1.67)			
△VR-12 physical								
Valgus	7.6 (3.5-11.7)	.398	5.4 (1.28-9.52)	.054	6.2 (2.16-10.24)	.221		
Neutral	10.7 (8.0-13.4)		10.97 (7.58-14.36)		9.8 (7-12.6)			
Varus	8.5 (3.8-13.2)		12.95 (7.76-18.14)		11.1 (7.13-15.07)			
△VR-12 mental								
Valgus	4.3 (2.2-6.4)	.141	2.2 (−2.07 to 6.47)	.889	1.3 (−1.92 to 4.52)	.587		
Neutral	0.7 (−1.4 to 2.8)		1.1 (−1.91 to 4.11)		−0.95 (−3.55 to 1.65)			
Varus	1.4 (−3.8 to 6.6)		2.3 (−3.93 to 8.53)		−0.2 (−4.54 to 4.14)			
△ KOOS symptom								
Valgus	6.8 (3.8-9.8)	.638	6.6 (3.59-9.61)	.365	5.7 (3.15-8.25)	.077		
Neutral	8.6 (6.2-11.0)		7.7 (5.42-9.98)		6.2 (4.28-8.12)			
Varus	7.9 (3.8-12.0)		10.2 (7.38-13.02)		11.6 (4.25-18.95)			
△ KOOS pain								
Valgus	16.4 (11.4-21.3)	.493	12.7 (6.42-18.98)	.053	15.8 (11.62-19.98)	**.022**	Neutal versus valgus	.49
Neutral	18.3 (13.6-23.0)		20.7 (15.75-25.65)		19.55 (15.78-23.32)		Varus versus valgus	**.016**
Varus	22.8 (18.2-27.4)		24.6 (16.23-32.97)		29.2 (16.66-41.74)		Varus versus neutral	.10
△ KOOS ADL								
Valgus	30.2 (22.0-38.4)	.712	23.5 (12.12-34.88)	.349	29.1 (21.08-37.12)	.390		
Neutral	34.5 (25.9-43.1)		34.6 (24.04-45.16)		36.7 (28.97-44.43)			
Varus	28.8 (20.4-37.2)		31.3 (18.11-44.49)		35.7 (23.78-47.62)			
△ KOOS-JR								
Valgus	24.0 (17.9-30.0)	.322	27 (0.27-53.73)	.249	25.1 (12.64-37.56)	.519		
Neutral	19.2 (10.2-28.2)		19.8 (11.81-27.79)		22.5 (14.06-30.94)			
Varus	30.5 (21.9-39.0)		32.7 (22.85-42.55)		31.5 (21.61-41.39)			
△KSS function								
Valgus	22.6 (16.4-28.8)	.581	17.8 (8.86-26.74)	.086	19 (12.24-25.76)	.316		
Neutral	21.4 (17.2-25.6)		19.7 (15.59-23.81)		21.2 (17.34-25.06)			
Varus	26.3 (18.7-33.9)		29.9 (22.78-37.02)		26.4 (18.64-34.16)			
△KSS symptom								
Valgus	11.7 (9.3-14.1)	.500	8.4 (5.38-11.42)	.208	10.2 (7.59-12.81)	.727		
Neutral	10.3 (8.5-12.1)		10.6 (8.91-12.29)		10.4 (8.78-12.02)			
Varus	12.1 (9.3-14.9)		11.9 (8.97-14.83)		11.7 (9.21-14.19)			
△KSS satisfaction								
Valgus	17.7 (14.6-20.8)	.856	13.8 (8.43-19.17)	.176	16.3 (12.47-20.13)	.472		
Neutral	18.0 (14.8-21.2)		18.5 (15.05-21.95)		18.2 (15.32-21.08)			
Varus	19.5 (16.2-22.8)		20.4 (14.79-26.01)		20.3 (15.36-25.24)			

ADL, activities of daily living; CI, confidence interval; KOOS, Knee injury and Osteoarthritis Outcome Score; KOOS-JR, Knee injury and Osteoarthritis Outcome Score for Joint Replacement; KSS, Knee Society Score; PROM, patient-reported outcome measure; UCLA, University of California Los Angeles Activity Scale; VR-12, Veterans RAND 12-Item Health Survey.

The bold was meant to highlight statistically significant values.

**Table 6:** Multivariate analysis of change from preoperative PROM.

ΔPROM	Compared to valgus	1-year follow-up		2-year follow-up		Final follow-up	
		Estimate (95% CI)	*P* value	Estimate (95% CI)	*P* value	Estimate (95% CI)	*P* value
△UCLA	Neutral	−0.4 (−1.2 to 0.3)	.285	0.1 (−0.8 to 1)	.850	0.42 (−0.37 to 1.2)	.295
	Varus	0.2 (−0.8 to 1.2)	.701	0.2 (−0.9 to 1.4)	.725	0.44 (−0.6 to 1.5)	.402
△VR-12 physical	Neutral	2.5 (−2.0 to 7.0)	.276	5.7 (0.5-11)	**.033**	2.8 (−1.9 to 7.5)	.238
	Varus	1.1 (−5.0 to 7.3)	.712	7.6 (0.7-14.4)	**.031**	4.5 (−1.7 to 10.7)	.155
△VR-12 mental	Neutral	−3.6 (−6.9 to −0.2)	**.037**	−0.6 (−5.3 to 4.2)	.809	−2.3 (−6.4 to 1.8)	.272
	Varus	−2.1 (−6.7 to 2.5)	.364	1 (−5.2 to 7.2)	.746	−1.2 (−6.6 to 4.2)	.662
△ KOOS symptom	Neutral	1.8 (−1.8 to 5.4)	.317	2.8 (−0.6 to 6.3)	.106	1.3 (−1.4 to 3.99)	.343
	Varus	0.6 (−5.2 to 6.5)	.831	3.7 (−0.8 to 8.3)	.106	2.3 (−1.7 to 6.3)	.248
△ KOOS pain	Neutral	2.8 (−3.8 to 9.4)	.396	10 (2-18)	**.015**	4.96 (−1.3 to 11.2)	.115
	Varus	6.2 (−4.4 to 16.8)	.248	11.6 (0.9-22.3)	**.034**	10.96 (2-19.9)	**.017**
△ KOOS ADL	Neutral	5.6 (−5.3 to 16.6)	.304	13.1 (−2 to 28.2)	.088	8.1 (−2.6 to 18.9)	.134
	Varus	3.4 (−14.6 to 21.3)	.707	11.5 (−9.5 to 32.6)	.275	9.3 (−6.1 to 24.7)	.229
△ KOOS-JR	Neutral	−5.3 (−21.2 to 10.5)	.501	−7.5 (−24.3 to 9.3)	.370	−3.2 (−19.98 to 13.6)	.703
	Varus	3.3 (−15.1 to 21.8)	.718	4 (−16 to 24.1)	.684	3.8 (−16.19 to 23.8)	.704
△KSS function	Neutral	−0.2 (−7.3 to 6.8)	.948	4.1 (−4.4 to 12.5)	.338	2.8 (−4.2 to 9.9)	.432
	Varus	2.8 (−6.8 to 12.4)	.565	12.5 (1.7-23.3)	**.024**	6.4 (−3 to 15.8)	.180
△KSS symptom	Neutral	−0.6 (−3.2 to 2.1)	.662	2.6 (−0.6 to 5.7)	.106	0.8 (−1.9 to 3.5)	.567
	Varus	0.7 (−2.8 to 4.3)	.684	3.4 (−0.7 to 7.4)	.103	1.5 (−2.1 to 5.1)	.409
△KSS satisfaction	Neutral	0.8 (−3.5 to 5.1)	.709	5.4 (−0.5 to 11.2)	.072	2.4 (−2.1 to 6.9)	.288
	Varus	2.0 (−3.8 to 7.8)	.490	6.9 (−0.7 to 14.5)	.075	4.3 (−1.7 to 10.2)	.161

ADL, activities of daily living; CI, confidence interval; KOOS, Knee injury and Osteoarthritis Outcome Score; KOOS-JR, Knee injury and Osteoarthritis Outcome Score for Joint Replacement; KSS, Knee Society Score; PROM, patient-reported outcome measure; UCLA, University of California Los Angeles Activity Scale; VR-12, Veterans RAND 12-Item Health Survey.

The bold was meant to highlight statistically significant values.

At final follow-up, univariate analyses found that patients in varus had significantly greater improvements in KOOS Pain scores compared to patients in residual valgus, with a mean change of 29.2 points versus 15.8 points, respectively (*P* = .016; Table 5). The multivariate model (Table 6) demonstrated that patients in postoperative varus alignment had significantly greater improvements in KOOS Pain scores by 10.96 points (*P* = .017; 95% CI: 2.0-19.9) at final follow-up, in comparison to valgus knees.

### Odds of achieving the minimal clinically important difference

At 1-year and 2-year follow-up, the odds of achieving the MCID for any PROM were not statistically different between postoperative alignment groups on univariate or multivariate analyses (Table 7; Table 8).

**Table 7:** Univariate analysis of achieving MCID postoperatively.

PROM per alignment group	1-year follow-up			2-year follow-up			Final follow-up		
	No MCID, N (%)	MCID, N (%)	*P* value	No MCID, N (%)	MCID, N (%)	*P* value	No MCID, N (%)	MCID, N (%)	*P* value
UCLA									
Valgus	13 (48)	14 (52)	.593	11 (52)	10 (48)	.606	16 (55)	13 (45)	.982
Neutral	31 (60)	21 (40)		19 (49)	20 (51)		31 (53)	27 (47)	
Varus	9 (60)	6 (40)		9 (64)	5 (36)		10 (56)	8 (44)	
VR-12 physical									
Valgus	12 (43)	16 (57)	.680	11 (44)	14 (56)	.127	15 (50)	15 (50)	.059
Neutral	17 (33)	34 (67)		10 (26)	29 (74)		19 (33)	38 (67)	
Varus	5 (33)	10 (67)		2 (14)	12 (86)		3 (17)	15 (83)	
VR-12 mental									
Valgus	18 (64)	10 (36)	.377	14 (56)	11 (44)	.783	23 (77)	7 (23)	.949
Neutral	40 (78)	11 (22)		25 (64)	14 (36)		45 (79)	12 (21)	
Varus	12 (80)	3 (20)		8 (57)	6 (43)		14 (78)	4 (22)	
KOOS symptom									
Valgus	13 (65)	7 (35)	.540	11 (58)	8 (42)	.378	16 (73)	6 (27)	.064
Neutral	12 (55)	10 (45)		12 (60)	8 (40)		21 (81)	5 (19)	
Varus	2 (40)	3 (60)		2 (29)	5 (71)		3 (38)	5 (63)	
KOOS pain									
Valgus	6 (30)	14 (70)	.352	7 (37)	12 (63)	.105	6 (27)	16 (73)	.585
Neutral	4 (18)	18 (82)		2 (10)	18 (90)		4 (15)	22 (85)	
Varus	0 (0)	5 (100)		1 (14)	6 (86)		1 (13)	7 (88)	
KOOS ADL									
Valgus	3 (15)	17 (85)	.809	6 (32)	13 (68)	.214	3 (14)	19 (86)	.349
Neutral	2 (9)	20 (91)		2 (10)	18 (90)		1 (4)	25 (96)	
Varus	0 (0)	5 (100)		2 (29)	5 (71)		1 (13)	7 (88)	
KOOS-JR									
Valgus	0 (0)	7 (100)	.206	1 (17)	5 (83)	.401	1 (13)	7 (88)	.667
Neutral	8 (31)	18 (69)		5 (28)	13 (72)		7 (24)	22 (76)	
Varus	1 (10)	9 (90)		0 (0)	7 (100)		1 (10)	9 (90)	
KSS function									
Valgus	5 (18)	23 (82)	.650	9 (36)	16 (64)	.105	9 (30)	21 (70)	.278
Neutral	10 (19)	42 (81)		7 (18)	32 (82)		9 (16)	49 (84)	
Varus	1 (7)	14 (93)		1 (7)	13 (93)		4 (22)	14 (78)	
KSS symptom									
Valgus	7 (25)	21 (75)	.497	12 (48)	13 (52)	.137	11 (37)	19 (63)	.316
Neutral	15 (29)	37 (71)		10 (26)	29 (74)		15 (26)	43 (74)	
Varus	2 (13)	13 (87)		3 (21)	11 (79)		3 (17)	15 (83)	
KSS satisfaction									
Valgus	2 (7)	26 (93)	.607	9 (36)	16 (64)	.105	6 (20)	24 (80)	.753
Neutral	8 (15)	44 (85)		7 (18)	32 (82)		8 (14)	50 (86)	
Varus	1 (7)	14 (93)		1 (7)	13 (93)		2 (11)	16 (89)	

ADL, activities of daily living; KOOS, Knee injury and Osteoarthritis Outcome Score; KOOS-JR, Knee injury and Osteoarthritis Outcome Score for Joint Replacement; KSS, Knee Society Score; MCID, minimal clinically important difference; UCLA, University of California Los Angeles Activity Scale; VR-12, Veterans RAND 12-Item Health Survey; PROM, patient reported outcome measure.

**Table 8:** Multivariate analysis of odds ratio for achieving MCID postoperatively.

PROM	Compared to valgus	1-year follow-up		2-year follow-up		Final follow-up	
		OR (95% CI)	*P* value	OR (95% CI)	*P* value	OR (95% CI)	*P* value
UCLA	Neutral	0.43 (0.15-1.22)	.118	0.99 (0.31-3.19)	.991	1.28 (0.44-3.88)	.653
	Varus	0.48 (0.11-2.04)	.326	0.55 (0.11-2.51)	.447	1.21 (0.3-4.93)	.792
VR-12 physical	Neutral	1.39 (0.52-3.69)	.509	2.28 (0.79-6.75)	.131	1.86 (0.73-4.8)	.191
	Varus	1.55 (0.42-6.27)	.520	4.71 (1-34.47)	.073	5.05 (1.28-26.02)	**.031**
VR-12 mental	Neutral	0.39 (0.11-1.29)	.124	0.76 (0.23-2.54)	.649	0.81 (0.26-2.64)	.721
	Varus	0.47 (0.07-2.48)	.395	1.23 (0.25-6.07)	.792	0.92 (0.19-4.03)	.916
KOOS symptom	Neutral	1.70 (0.45-6.94)	.441	1.81 (0.42-8.9)	.440	1.15 (0.3-4.46)	.839
	Varus	2.69 (0.34-26.02)	.352	4.31 (0.63-40.89)	.155	2.21 (0.33-14.92)	.416
KOOS pain	Neutral	1.96 (0.46-9.10)	.368	5.6 (1.08-43.82)	.058	3.41 (0.68-17.22)	.137
	Varus	-	-	3.47 (0.45-72.68)	.293	3.56 (0.27-47.23)	.336
KOOS ADL	Neutral	1.66 (0.24-13.94)	.605	4.31 (0.83-33.3)	.105	1.49 (0.5-4.39)	.473
	Varus	-	-	1.23 (0.19-10.67)	.834	1.43 (0.27-7.53)	.671
KOOS-JR	Varus vs Neutral	2.44 (0.29-52.14)	.456	0.52 (0.02-4.44)	.590	0.74 (0.1-5.58)	.771
				-	-	2.94 (0.22-38.86)	.413
KSS function	Neutral	0.97 (0.27-3.19)	.956	2.96 (0.91-10.17)	.075	2.92 (1.1-7.79)	**.032**
	Varus	2.64 (0.36-54.16)	.403	7.43 (1.15-147.23)	.074	2.84 (0.74-10.99)	.130
KSS symptom	Neutral	0.98 (0.31-2.93)	.966	2.9 (0.99-8.87)	.055	2.78 (0.69-11.23)	.152
	Varus	2.38 (0.46-18.43)	.337	3.37 (0.81-17.83)	.114	3.86 (0.6-25.03)	.157
KSS satisfaction	Neutral	0.41 (0.06-1.89)	.296	2.52 (0.8-8.32)	.118	1.75 (0.19-15.64)	.619
	Varus	0.94 (0.08-21.78)	.962	7.26 (1.14-142.59)	.076	1.83 (0.1-33.78)	.686

ADL, activities of daily living; CI, confidence interval; KOOS, Knee injury and Osteoarthritis Outcome Score; KOOS-JR, Knee injury and Osteoarthritis Outcome Score for Joint Replacement; KSS, Knee Society Score; MCID, minimal clinically important difference; OR, odds ratio; UCLA, University of California Los Angeles Activity Scale; VR-12, Veterans RAND 12-Item Health Survey; PROM, patient reported outcome measure.

The bold was meant to highlight statistically significant values.

When assessing final follow-up, the univariate model (Table 7) found no significant differences between alignment groups. On multivariate analyses (Table 8), patients in varus alignment had 5.05 higher odds of achieving the MCID for VR-12 Physical scores than patients in valgus (95% CI: 1.28-26.02; *P* = .031). Patients in neutral alignment were 2.92 times more likely to achieve MCID on KSS Function scores than patients in valgus alignment (95% CI: 1.10-7.79; *P* = .032).

## Discussion

In this study, we investigated whether postoperative mechanical alignment influences clinical outcomes in patients undergoing TKA for osteoarthritis in the setting of preoperative valgus deformities. Failure can be twice as high in patients undergoing TKA for valgus alignment, highlighting the increased technical challenges in these patients [18].

Some surgeons propose that retaining some valgus alignment allows for a kinematic-friendly approach to balancing. With this approach, it is believed that patients may benefit from soft-tissue feedback as they maintain their accustomed alignment [19,20]. Moreover, leaving patients in residual valgus allows for a technically less challenging surgery than correction into neutral or varus, as the lateral structures do not have to be released as extensively. Yet, the placement of components in excessive varus or valgus leads to higher rates of failure, likely influenced by increased stress at the bone-implant interface [21]. However, the impact of minor variations of postoperative coronal alignment is poorly understood [7,22].

In this study, we used 2° of deviation from the mechanical axis as the cutoff for alignment groups. While a cutoff of 3° has been historically used in the literature, this number emanates from early studies demonstrating higher rates of mechanical loosening after TKA in patients with postoperative coronal plane alignment more than 3° from neutral [23]. While revision rates were evaluated in this study, the primary outcome was PROMs. We could not identify robust evidence on the 3° cutoff when assessing patient-reported outcomes. With modern implantation techniques (eg, navigation, robotics) leading to increasing precision, a 2° cutoff represented a more practical and modern evaluation criterion.

Postoperative valgus alignment outperformed neutral or varus alignment only once on multivariate analysis of VR-12 Mental at 1-year follow-up. Although statistically significant, the 3.6-point difference falls below the established MCID of 5.99 points, suggesting it is unlikely to be clinically meaningful. The key finding of this study is that multiple PROMs assessing pain and function were superior when patients were placed into a varus or neutral mechanical alignment postoperatively. We postulate that some of the differences in outcomes may be due to soft-tissue factors. Knees with residual valgus due to deformity undercorrection have continued loading of chronically loose medial collateral structures, which may contribute to instability and pain. This may also explain the improved pain, function, and satisfaction scores reported in patients corrected to neutral and varus alignment [24,25].

While our results indicate worse outcomes in postoperative valgus knees, studies suggest preoperative varus alignment may be more tolerant to variations in final coronal alignment. Meneghini et al. studied 176 consecutive patients in preoperative varus undergoing TKA by a single surgeon between 2010 and 2014 with at least 12 months of follow-up. Postoperatively, 45 patients were in residual varus, 116 in neutral, and 15 were in valgus based on anteroposterior weight-bearing knee radiographs [26]. The authors found no differences in absolute or delta KSS and UCLA scores among postoperative alignment groups. However, the use of short-knee radiographs rather than 36-inch long-leg radiographs may have compromised the accuracy of the author’s alignment measurements.

Our study also demonstrated that patients continued to have improvements in PROMs more than 2 years postoperatively. Other studies assessing subjective outcomes after TKA have shown that satisfaction is not static over time. Thus, identifying factors that influence long-term changes in satisfaction is critical to creating durable outcomes [[27], [28], [29]]. A recent study by Blackburn et al. analyzed 402 patients with preoperative varus or valgus alignment who underwent TKA. The authors found that satisfaction scores changed among 1, 3, and 5 years postoperatively. Furthermore, higher scores in multiple PROMs at 1 year postoperatively predicted better outcomes at later time points [27]. Our results support the idea that longer-term follow-up is needed to fully assess satisfaction after TKA, as we found that patients in residual valgus postoperatively saw less improvement on KOOS Pain scores and had lower odds of reaching MCID on VR-12 Physical and KSS Function scores more than 2 years postoperatively.

This study has several limitations. First, it is a retrospective design and lacks randomization. Fortunately, demographic data for each alignment group were similar and did not indicate any confounding variables in our model. Yet, it is possible that unmeasured confounding factors influenced the results. Additionally, this study evaluates coronal plane alignment but does not analyze the sagittal or axial planes, which may affect soft-tissue tension and patient outcomes [30]. Next, while all surgeons used a gap-balancing technique, variations in the specific methods, implant selection, and other subtle differences in intraoperative practices may have influenced the outcomes. Other techniques may yield different results (eg, planned measured resection with a neutral target), but outliers exist nonetheless. Additionally, differences in the level of constraint used and the final alignment achieved by each surgeon could have further impacted the results. We did not have long-term follow-up on all eligible patients, particularly after 5 years as only 20.8% of patients had follow-up after this time point. Finally, only a subset of the PROMs analyzed in this study was found to be significant in the results. For example, the VR-12 Physical and KSS Function scores were significant and evaluate functional characteristics such as climbing stairs, walking, and doing household chores. However, the KOOS activities of daily living, which evaluates similar activities, was not. Similarly, KOOS Pain was significant, while KSS Symptoms was not. The responsiveness of a PROM is its ability to detect change over time, and there are known differences in responsiveness between different PROMs used in evaluating patients undergoing TKA [[31], [32], [33]]. It may be that the PROMs we found to be significant in this study were worded such that they were able to detect subtle differences in this population, while others were not. Finally, the relative rarity of valgus preoperative deformities and reliance on PROMs make this a difficult population to study. The power of the present study was limited by the sample size within each of the postoperative alignment groups—30 patients in valgus, 58 in neutral, and 18 in varus. With these numbers, the study was 80% powered to detect the following differences in PROMs for patients in neutral and varus when compared to residual valgus: 1.6 points for neutral and 2.2 points for varus on the UCLA, 15.2 points for neutral and 22.1 points for varus on the KOOS subscales, 7.9 points for neutral and 10.6 points for varus on the KSS subscales, and 7.6 points for neutral and 10.1 points for varus on the VR-12 subscales. The significantly larger improvements in KOOS Pain at final follow-up and KSS scores at 2-year follow-up for varus versus valgus alignment exceeded the minimum detectable thresholds of our study’s power. Despite these limitations, this study provides important insight for surgeons attempting to optimize postoperative coronal alignment in patients with preoperative valgus deformities undergoing TKA.

## Conclusions

Patients with preoperative valgus deformities undergoing TKA experienced improvements in PROMs regardless of final alignment. Correction to varus or neutral postoperative alignment was associated with greater improvements in PROMs, as well as higher odds of achieving the MCID of these PROMs when compared to knees in residual valgus. This difference was first observed 2 years postoperatively and persisted in several PROMs at the final follow-up. Care must be taken in patients with preoperative valgus alignment, as this study indicates that clinical outcomes with residual valgus may be inferior to those seen with postoperative varus or neutral alignment. Future studies with larger patient populations, using models to evaluate all radiographic parameters and intraoperative techniques, could be useful to control for additional factors that influence PROMs.

## Conflicts of interest

William J. Maloney received royalties from Stryker and Zimmer Biomet; holds stock or stock options in TJO; received royalties, financial or material support from Wolters Kluwer; and is the President of The Knee Society. James I. Huddleston received royalties from Exactech and DePuy; is a paid consultant for Exactech and DePuy; holds stock or stock options in Corin and Porosteon; received research support from Apple and Zimmer Biomet as a Principal Investigator; received royalties, financial or material support from Wolters Kluwer; and is a board member of AAOS, AJRR, AAHKS, Knee Society, and Hip Society. All other authors declare no potential conflicts of interest.

For full disclosure statements refer to https://doi.org/10.1016/j.artd.2025.101660.

## CRediT authorship contribution statement

**Anastasia A. Hunt:** Writing – review & editing, Writing – original draft, Methodology, Investigation, Data curation. **Ian Hollyer:** Writing – review & editing, Writing – original draft, Data curation. **Nicole S. Pham:** Writing – review & editing, Formal analysis. **William J. Maloney:** Writing – review & editing, Supervision. **James I. Huddleston:** Writing – review & editing, Supervision, Conceptualization.

## Appendix ASupplementary data

Conflict of Interest Statement for Hunt

Conflict of Interest Statement for Hollyer

Conflict of Interest Statement for Huddlesto

Conflict of Interest Statement for Pham

Conflict of Interest Statement for Maloney

## References

[1] Jauregui J.J., Cherian J.J., Pierce T.P., Beaver W.B., Issa K., Mont M.A. (2015). Long-term survivorship and clinical outcomes following total knee arthroplasty. J Arthroplast.

[2] Lange J., Haas S.B. (2017). Correcting severe valgus deformity: taking out the knock. Bone Joint J.

[3] Abhari S., Hsing T.M., Malkani M.M., Smith A.F., Smith L.S., Mont M.A. (2021). Patient satisfaction following total knee arthroplasty using restricted kinematic alignment. Bone Joint J.

[4] Nam D., Nunley R.M., Barrack R.L. (2014). Patient dissatisfaction following total knee replacement: a growing concern?. Bone JointJ.

[5] (2022). There is no difference in composite/functional outcomes or complications between kinematic or mechanical alignment principles in total knee arthroplasty. Am Acad Orthop Surg.

[6] Insall J.N., Binazzi R., Soudry M., Mestriner L.A. (1985). Total knee arthroplasty. Clin Orthop Relat Res.

[7] Ritter M.A., Davis K.E., Davis P., Farris A., Malinzak R.A., Berend M.E. (2013). Preoperative malalignment increases risk of failure after total knee arthroplasty. J Bone Joint Surg Am.

[8] Lee S.S., Lee H., Lee D.H., Moon Y.W. (2018). Slight under-correction following total knee arthroplasty for a valgus knee results in similar clinical outcomes. Arch Orthop Trauma Surg.

[9] Lizaur-Utrilla A., Serna-Berna R., Vizcaya-Moreno M.F., Martinez-Mendez D., Marco-Gomez L., Lopez-Prats F.A. (2018). Comparison of functional outcomes between the first and second knee in staged bilateral total knee arthroplasty with diverse intervals between stages. J Arthroplast.

[10] Lizaur-Utrilla A., Gonzalez-Parreño S., Martinez-Mendez D., Miralles-Muñoz F.A., Lopez-Prats F.A. (2020). Minimal clinically important differences and substantial clinical benefits for Knee Society Scores. Knee Surg Sports Traumatol Arthrosc.

[11] Shichman I., Oakley C.T., Konopka J.A., Ashkenazi I., Rozell J., Schwarzkopf R. (2023). The association of metabolic syndrome on complications and implant survivorship in primary total knee arthroplasty in morbidly obese patients. J Arthroplast.

[12] Chalmers B.P., Puri S., Chiu Y.-F., Lebowitz J., Sideris A., Westrich G.H. (2023). Patients undergoing primary, cementless TKA had similar pain, opioid utilization, and functional outcomes compared to matched patients with cemented fixation. J Arthroplast.

[13] Zhou L., Natarajan M., Miller B.S., Gagnier J.J. (2018). Establishing minimal important differences for the VR-12 and SANE scores in patients following treatment of rotator cuff tears. Orthop J Sports Med.

[14] Roos E.M., Lohmander L.S. (2003). The Knee injury and Osteoarthritis Outcome Score (KOOS): from joint injury to osteoarthritis. Health Qual Life Outcome.

[15] Hung M., Bounsanga J., Voss M.W., Saltzman C.L. (2018). Establishing minimum clinically important difference values for the Patient-Reported Outcomes Measurement Information System Physical Function, hip disability and osteoarthritis outcome score for joint reconstruction, and knee injury and osteoarthritis outcome score for joint reconstruction in orthopaedics. World J Orthop.

[16] SooHoo N.F., Li Z., Chenok K.E., Bozic K.J. (2015). Responsiveness of patient reported outcome measures in total joint arthroplasty patients. J Arthroplast.

[17] Copay A.G., Eyberg B., Chung A.S., Zurcher K.S., Chutkan N., Spangehl M.J. (2018). Minimum clinically important difference: current trends in the orthopaedic literature, Part II: lower extremity: a systematic review. JBJS Rev.

[18] Mazzotti A., Perna F., Golinelli D., Quattrini I., Stea S., Bordini B. (2019). Preoperative valgus deformity has twice the risk of failure as compared to varus deformity after total knee arthroplasty. Knee Surg Sports Traumatol Arthrosc.

[19] Waterson H.B., Clement N.D., Eyres K.S., Mandalia V.I., Toms A.D. (2016). The early outcome of kinematic versus mechanical alignment in total knee arthroplasty: a prospective randomised control trial. Bone JointJ.

[20] Slevin O., Hirschmann A., Schiapparelli F.F., Amsler F., Huegli R.W., Hirschmann M.T. (2018). Neutral alignment leads to higher knee society scores after total knee arthroplasty in preoperatively non-varus patients: a prospective clinical study using 3D-CT. Knee Surg Sports Traumatol Arthrosc.

[21] Cherian J.J., Kapadia B.H., Banerjee S., Jauregui J.J., Issa K., Mont M.A. (2014). Mechanical, anatomical, and kinematic axis in TKA: concepts and practical applications. Curr Rev Musculoskelet Med.

[22] Ritter M.A., Davis K.E., Meding J.B., Pierson J.L., Berend M.E., Malinzak R.A. (2011). The effect of alignment and BMI on failure of total knee replacement. J Bone Joint Surg Am.

[23] Jeffery R., Morris R., Denham R. (1991). Coronal alignment after total knee replacement. J Bone Joint Surg Br.

[24] Hamahashi K., Mitani G., Takagaki T., Serigano K., Mochida J., Sato M. (2016). Clinical outcomes of patients with valgus deformity undergoing minimally invasive total knee arthroplasty through the medial approach. Open Orthop J.

[25] Howell S.M., Shelton T.J., Hull M.L. (2018). Implant survival and function ten years after kinematically aligned total knee arthroplasty. J Arthroplast.

[26] Meneghini R.M., Grant T.W., Ishmael M.K., Ziemba-Davis M. (2017). Leaving residual varus alignment after total knee arthroplasty does not improve patient outcomes. J Arthroplast.

[27] Blackburn A.Z., Feder O., Amakiri I., Melnic C.M., Huddleston I.I.I.J.I., Malchau H. (2023). One-year postoperative patient-reported outcome measures are associated with three-year to five-year postoperative satisfaction in total knee arthroplasty. J Arthroplast.

[28] Clement N., Bardgett M., Weir D., Holland J., Gerrand C., Deehan D. (2018). Three groups of dissatisfied patients exist after total knee arthroplasty: early, persistent, and late. Bone Joint J.

[29] Galea V.P., Rojanasopondist P., Connelly J.W., Bragdon C.R., Huddleston I.I.I.J.I., Ingelsrud L.H. (2020). Changes in patient satisfaction following total joint arthroplasty. J Arthroplast.

[30] Rahm S., Camenzind R.S., Hingsammer A., Lenz C., Bauer D.E., Farshad M. (2017). Postoperative alignment of TKA in patients with severe preoperative varus or valgus deformity: is there a difference between surgical techniques?. BMC Musculoskelet Disord.

[31] Hung M., Saltzman C.L., Greene T., Voss M.W., Bounsanga J., Gu Y. (2018). Evaluating instrument responsiveness in joint function: the HOOS JR, the KOOS JR, and the PROMIS PF CAT. J Orthop Res.

[32] Davis A., Perruccio A., Canizares M., Hawker G., Roos E., Maillefert J.-F. (2009). Comparative, validity and responsiveness of the HOOS-PS and KOOS-PS to the WOMAC physical function subscale in total joint replacement for osteoarthritis. Osteoarthr Cartil.

[33] Ramkumar P., Harris J.D., Noble P. (2015). Patient-reported outcome measures after total knee arthroplasty: a systematic review. Bone Joint Res.

