# Supplementary material for: Residual Postoperative Valgus After Total Knee Arthroplasty for Preoperative Valgus Deformity Results in Inferior Patient-Reported Outcomes
Source: Arthroplast Today. 2025 Mar 13;32:101660. doi: 10.1016/j.artd.2025.101660 (PMC11954113; doi:10.1016/j.artd.2025.101660)
Supplement: Conflict of Interest Statement for Maloney [file mmc5.pdf]

# INDIVIDUAL CONFLICT OF INTEREST STATEMENT

## *American Association of Hip and Knee Surgeons*

(Adopted from the American Academy of Orthopaedic Surgeons disclosure statement)

The following form **must be filled out completely and submitted by each author (example, 6 authors, 6 forms). All items require a response. If there is no relevant disclosure for a given item, enter "*None.*"**

---

**Manuscript Title** Residual Postoperative Valgus after Total Knee Arthroplasty for Preoperative Valgus Deformity Results in Inferior Patient Reported Outcomes

1. Royalties from a company or supplier (The following conflicts were disclosed)
   Stryker
   Zimmer

2. Speakers bureau/paid presentations for a company or supplier (The following conflicts were disclosed)

   N/A

3A. Paid employee for a company or supplier (The following conflicts were disclosed)

   N/A

3B. Paid consultant for a company or supplier (The following conflicts were disclosed)
   N/A

3C. Unpaid consultants for a company or supplier (The following conflicts were disclosed)

   N/A

4. Stock or stock options in a company or supplier (The following conflicts were disclosed)

   TJO

5. Research support from a company or supplier as a Principal Investigator (The following conflicts were disclosed)

   N/A

6. Other financial or material support from a company or supplier (The following conflicts were disclosed)

   N/A

7. Royalties, financial or material support from publishers (The following conflicts were disclosed)

   Wolters Kluwer

8. Medical/Orthopaedic publications editorial/governing board (The following conflicts were disclosed)

   N/A

9. Board member/committee appointments for a society (The following conflicts were disclosed)

   The Knee Society - President

**Each author must sign AND print or type his/her name, date and submit a separate form**

In addition, one BLINDED Conflict of Interest form (no author names used) should be submitted per manuscript with all author disclosures.

---

| | | |
|---|---|---|
| William J. Maloney | | Jan. 10, 2024 |
| Author Name (Print or Type) | Author Signature | Date |
